# Clinical metabolomics: Useful insights, perspectives and challenges

**DOI:** 10.1016/j.metop.2024.100290

**Published:** 2024-05-31

**Authors:** Maria Dalamaga

**Affiliations:** Department of Biological Chemistry, School of Medicine, National and Kapodistrian University of Athens, 75 Mikras Asias, 11527, Athens, Greece

**Keywords:** Mass spectrometry, Metabolite, Metabolomics, Multi-omics, Obesity, Omics, Precision medicine

## Abstract

Metabolomics, a cutting-edge omics technique, is a rapidly advancing field in biomedical research, concentrating on the elucidation of pathogenetic mechanisms and the discovery of novel metabolite signatures predictive of disease risk, aiding in earlier disease detection, prognosis and prediction of treatment response. The capacity of this omics approach to simultaneously quantify thousands of metabolites, i.e. small molecules less than 1500 Da in samples, positions it as a promising tool for research and clinical applications in personalized medicine. Clinical metabolomics studies have proven valuable in understanding cardiometabolic disorders, potentially uncovering diagnostic biomarkers predictive of disease risk. Liquid chromatography-mass spectrometry is the predominant analytical method used in metabolomics, particularly untargeted. Metabolomics combined with extensive genomic data, proteomics, clinical chemistry data, imaging, health records, and other pertinent health-related data may yield significant advances beneficial for both public health initiatives, clinical applications and precision medicine, particularly in rare disorders and multimorbidity. This special issue has gathered original research articles in topics related to clinical metabolomics as well as research articles, reviews, perspectives and highlights in the broader field of translational and clinical metabolic research. Additional research is necessary to identify which metabolites consistently enhance clinical risk prediction across various populations and are causally linked to disease progression.

## 1 Introduction to clinical metabolomics

Precision medicine aims to enhance patient prognosis and stratification by leveraging knowledge of biological mechanisms and biomarkers [1]. Research and practices oriented towards precision medicine have been successfully implemented in various diseases, including metabolic, malignant, respiratory, cardiovascular, digestive tract and inflammatory. The applications of precision medicine can be further categorized into several areas, such as large-scale screening, early detection, molecular phenotyping and prognosis of disease as well as prediction of treatment response [1].

Metabolomics, a cutting-edge omics technique, has emerged due to the advance in analytical methods and bioinformatics. Typically, the metabolomics approach utilizes sophisticated analytical chemistry tools, such as nuclear magnetic resonance (NMR) and mass spectrometry (MS) in combination with various chromatographic techniques, i.e. gas chromatography (GC-MS) or liquid chromatography (LC-MS), to determine and characterize the metabolome, focusing on molecules smaller than 1500 Da in cells, organs, tissues, or biofluids [2]. The recent emergence of medical genomics introduces innovative technological tools for determining genetic predispositions to diseases. However, metabolomics enables us to go further by connecting the pathological phenotype with gene expression defects and disorders.

The term "clinical metabolomics" first appeared in the literature in 2009, although the concept was initially described in 2008 [3]. The objective of clinical metabolomics is to assess and predict a subject's health and disease risk by determining metabolic signatures in body fluids (plasma, urine, saliva, cerebrospinal fluid, etc) or tissues, which are influenced by genetics, epigenetics, dietary patterns, environmental parameters and behavior [4]. Metabolic signatures comprise a group or a combination of affected metabolites.

The metabolome may be studied both intracellularly, revealing functional abnormalities at the cellular level, and peripherally, such as in biofluids, particularly blood plasma, which contains a multitude of metabolites that reflect organ metabolic activity and provide valuable pathophysiological information [2]. Some metabolites in human plasma are synthesized endogenously, while others originate from the environment. The variability in the metabolome is partly hereditary and is significantly influenced by parameters such as dietary patterns, gut microbiota and lifestyle habits (e.g., smoking or physical activity). Diet and the microbiome constitute the main contributors to individual metabolic variability, rendering each person's metabotype unique [5]. However, in the absence of significant health changes, an individual's metabotype remains relatively stable throughout time.

The examination of metabolites is based on two general approaches: 1) targeted metabolomics, which focuses on determining a specific set of metabolites, and 2) untargeted metabolomics, which uses an unbiased approach to determine and compare as many metabolites as possible, comprising unknown ones, between samples. Using the latter approach, a plethora of metabolite features—peaks corresponding to individual ions with distinctive mass-to-charge (*m*/*z*) ratios and retention times (RT)—may be routinely detected via LC/MS-based methods [2]. The choice between these approaches depends on the specific desired application.

The two primary techniques for metabolomics studies are mass spectrometry (MS)-based metabolomics and nuclear magnetic resonance (NMR) spectroscopy. Combining these methods can address many of their individual limitations, thereby enhancing metabolome coverage [6]. MS-based metabolomics provides a wide metabolite coverage ranging from polar metabolites to non-polar lipids and excels at detecting low-abundance metabolites, whilst NMR is effective at identifying core metabolites in key metabolic pathways [7]. Moreover, NMR is analytically more powerful, high-throughput and low cost, allowing the study of many participants [8]. Notwithstanding untargeted metabolomics has influenced the pathogenetic understanding of disease pathways via the identification of novel biomarkers in metabolic pathways, it could detect metabolites that may not be identified because they are not present in the software library [9].

Metabolomics studies have proven valuable in understanding cardiometabolic disorders, potentially uncovering diagnostic biomarkers predictive of disease risk. This could facilitate earlier disease detection and improve prognosis. Obesity increases the risk of various health conditions, including cardiovascular disease, type 2 diabetes (T2DM), cancer, infections and autoimmune disorders [[10], [11], [12], [13], [14], [15]]. However, the mechanisms connecting adiposity to adverse health outcomes remain not fully elucidated, whereas the metabolome may play an important role. In obesity research, metabolomics has been utilized to predict body mass index (BMI) and adiposity, enhance the understanding of metabolic dysregulation caused by increased adiposity, and forecast the effectiveness of obesity treatment [16]. Metabolic disturbances stemming from increased adiposity are systemic and far-reaching, comprising glucose and lipid metabolism and being implicated in low-grade chronic inflammation. Robust associations have consistently been seen with branched-chain amino acids (BCAAs) [16]. An untargeted metabolomics study revealed that nearly a third of the metabolome is linked to BMI, specifically lipids, amino acids, and peptides. The majority of these metabolites, including glucose, alanine, tyrosine, kynurenate, γ-glutamyltyrosine and phospholipids, are also linked to higher insulin resistance and may mediate the connection between adiposity and metabolic disorders [17]. Characterizing metabolic profiles of adiposity (and fat distribution) can enhance its clinical management. Metabolomics studies have identified that plasma steroid sulfates and amino acids are indicative of visceral and subcutaneous adiposity in subjects with obesity who do not have insulin resistance [18]. Larger clinical metabolomics studies may shed light on the pathways characterizing metabolically unhealthy overweight and obesity, leading to more effective treatments.

T2DM is an increasingly prevalent metabolic disorder linked to complications such as cardiovascular and renal diseases as well as retinopathy and infections [[19], [20], [21]]. Among the metabolite classes associated with T2DM, BCAAs are particularly prominent, with significant associations consistently shown in large clinical metabolomics studies [22]. Clinical metabolomics may illuminate pathways and biomarkers that predict the progression from normoglycemia to T2DM, from prediabetes to T2DM and the development of diabetic complications. In subjects with normal fasting glucose, data from a specific set of 19 metabolites enhanced the prediction of T2DM beyond conventional risk factors. Additionally, the nitrogen metabolism pathway, which comprises 3 prioritized metabolites, i.e. glycine, taurine and phenylalanine, and its components were identified as potential effectors in T2DM onset [23]. In a meta-analysis, metabolite concentrations of alanine, glutamate, and palmitic acid (C16:0) were significantly different between prediabetic and diabetic patients [24]. In another meta-analysis of clinical metabolomics studies, 5 metabolites, characterized as essential metabolites, differed significantly in patients with diabetic renal disease (decreased glycine, aconitic acid, glycolic acid and uracil, and increased cysteine) compared to controls [25]. Further research is required to validate these findings and assess their clinical meaning.

Finally, research that integrates genomics with comprehensive metabolic profiling using high-throughput metabolomics platforms has facilitated the discovery of numerous loci associated with metabolic traits related to circulating lipids, lipoproteins, fatty acids, and amino acids. These investigations offer fresh insights into human metabolism biology, and have steered large epidemiological studies, such as Mendelian randomization analyses, to infer causal associations [26]. In the very recent and largest metabolomics study combined with genome-wide association study involving 136,016 participants, over 400 independent genomic regions influencing 233 circulating metabolic biomarkers, including lipoproteins and lipids, were identified [27]. Genetic pleiotropy, whereas the same genetic variation influences several metabolic processes, spanning traits related to lipids, lipoproteins, and fatty acids, was an important study finding. This study also underscored the significance of participant characteristics, such as fasting status, which can significantly influence genetic associations [27].

Metabolomics combined with extensive genomic data, proteomics, clinical chemistry data, imaging, health records, and other pertinent health-related data may yield significant discoveries beneficial for both public health initiatives, clinical applications and precision medicine, particularly in rare disorders and multimorbidity [28]. This multi-omics approach combined with health and laboratory data is advancing with the integration of machine learning tools and artificial intelligence. The generated extensive data enable the training of robust machine learning models for both classification and regression analyses. In metabolomics, additional research is necessary to determine which metabolites consistently enhance clinical risk prediction across diverse populations and are causally linked to disease progression. Besides the hurdles associated with replication issues, the development of validated assays and the enhancement of study participants diversity may enhance the clinical relevance of findings in metabolomics studies [29]. These challenges encompass the identification of the chemical composition of unidentified metabolites arising from untargeted metabolomics, the standardization of metabolites across various platforms and experiments, the decrease of expenses related to untargeted metabolomics, and the thorough modeling disease risk in integrative multi-omics investigations.

## 2 Special issue on clinical metabolomics

This special issue has gathered original research articles in topics related to clinical metabolomics as well as research articles, reviews, perspectives and highlights in the broader field of translational and clinical metabolic research.

Alzheimer's Disease (AD) is a complex, multifactorial condition that requires innovative approaches to uncover its underlying mechanisms. Metabolites, which comprise the final products of genes, transcripts, and proteins regulations, may reveal insights into disease pathogenesis [30]. While blood is commonly used in metabolomics studies, extracellular vesicles (EVs), which contain cell-specific biological material and can cross the blood-brain barrier, merit further exploration as a potential source of biological material [[30], [31], [32]]. Nielsen et al. aimed at examining metabolites derived from blood and EVs to better understand the pathogenetic mechanisms of AD [32]. Although no significant EV-derived metabolites were identified that could differentiate patients from healthy individuals, six important serum metabolites were discovered: valine, histidine, allopurinol riboside, inosine, 4-pyridoxic acid, and guanosine. Pathway analysis indicated that BCAAs, purine, and histidine metabolism were downregulated, while vitamin B6 metabolism was upregulated in AD patients compared to controls [32]. Using a combination of LC-MS and NMR methodologies, the study identified several altered mechanisms potentially related to AD pathogenesis. The researchers concluded that further optimization of EVs is necessary before they can be effectively used as a biological material for AD-related metabolomics studies [32].

Small cell lung cancer (SCLC) is a malignant disease with a poor prognosis, with most patients being already in a metastatic stage at diagnosis. Current diagnostic methods rely on clinical imaging using computed tomography (CT) and positron emission tomography/CT in combination with cytological/histopathological biopsies of the tumor-suspected lesions of the lung [33,34] Few blood-based biomarkers have been clinically examined for diagnosis and screening [[35], [36], [37], [38], [39], [40]]. Although the precise pathogenetic mechanisms of SCLC are not fully understood, several genetic mutations, protein and metabolic abnormalities have been identified. The study by Pedersen et al. aimed at identifying metabolite alterations related to SCLC in order to enhance the understanding of this aggressive cancer [40]. Among others, metabolites involved in tricarboxylic acid cycle (succinate), lipid metabolism (LDL triglyceride, LDL-1 triglyceride, LDL-2 triglyceride, LDL-6 triglyceride, LDL-4 cholesterol, HDL-3 free cholesterol, HDL-4 cholesterol, HDL-4 apolipoprotein-A1, HDL-4 apolipoprotein-A2), amino acids (glutamic acid, glutamine, leucine, isoleucine, valine, lysine, methionine, tyrosine, creatine), and ketone body metabolism (3-hydroxybutyric acid, acetone) were found deranged in the pre-treatment serum samples of SCLC patients compared to healthy individuals. This study offers new insights into the metabolic disturbances in pre-treatment SCLC patients, enhancing the molecular understanding of this malignant disease [40].

Research has demonstrated that cell metabolism actively influences the regulation of stemness and fate determination. The study by Olesen et al. aimed to characterize the metabolic activity of mesenchymal stromal cells (MSCs) from various developmental stages in response to different oxygen levels during expansion, with the goal of identifying optimal culture conditions for MSCs before transplantation [41]. In contrast to adult MSCs, they found that fetal MSCs exhibited similar respiration and aerobic glycolysis across various oxygen culture concentrations during expansion. Adult MSCs adapted their respiration to glycolytic activities based on the culture conditions, indicating a more mature metabolic function. These observations are pertinent for establishing ideal in vitro culture conditions to enhance engraftment and therapeutic efficacy [41].

The gut mycobiome, although small, is a crucial and functional component of the gut ecosystem. Changes in its composition have been linked to several diseases, including colorectal cancer, inflammatory bowel disease, and irritable bowel syndrome [[42], [43], [44], [45]]. However, little is known about the composition and long-term stability of the gut mycobiome in middle age and later life, as well as the interactions between gut fungal and bacterial communities in maintaining metabolic homeostasis. Additionally, the relationship between the gut mycobiome and metabolic health is not well understood. The gut mycobiome has been associated with cardiometabolic disorders in animal models and some human studies [44]. The perspective by Dalamaga et al. reviewed the findings of a multiomics and longitudinal study conducted by Dr. Zheng and colleagues [44,46]. This study explored the impact of age, diet, and various sociodemographic and clinical factors on the gut mycobiome. The research involved mapping the gut mycobiome of 1244 Chinese middle-aged and elderly adults from the population-based Guangzhou Nutrition and Health Study cohort [46]. The study findings underscore the significance of the intestinal fungal community as a crucial element within the gut ecosystem, contributing significantly to its long-term stability. This integrated cross-kingdom analysis has the potential to expand our understanding and identification of new preventive and therapeutic targets for metabolic disorders [46].

Sepsis, characterized by life-threatening organ dysfunction resulting from infection, represents a significant global cause of mortality and morbidity [[47], [48], [49], [50]]. Given its systemic nature, multiple organ systems may become affected [[51], [52], [53]]. During sepsis, impaired liver function correlates with elevated bilirubin levels and reduced levels of fetuin-A, a significant hepatokine [[54], [55], [56]]. Karampela et al. sought to investigate the early association between bilirubin to fetuin-A (B/F) ratio in sepsis and its impact on severity and prognosis among critically ill patients [56]. The baseline and one-week B/F ratios exhibited a notably higher level in individuals experiencing septic shock and those who did not survive, in contrast to those with sepsis and survivors. Additionally, the B/F ratio displayed a positive correlation with severity scores and demonstrated superior predictive capability for mortality compared to bilirubin in ROC curve analysis. These findings suggest that the B/F ratio may hold promise as a potential sepsis biomarker with predictive utility in critically ill patients [56].

Obesity and insulin resistance are increasingly prevalent among individuals with Type 1 Diabetes Mellitus (T1DM) [[57], [58], [59]]. Genetic and epigenetic factors, along with subcutaneous insulin administration, are implicated in the development of this concurrent condition [59]. Growing evidence suggests that the co-occurrence of obesity and IR independently predicts worse cardiovascular disease (CVD) outcomes. In an integrative literature review, Sikorskaya et al. aimed to evaluate the efficacy and safety of metformin as an add-on therapy to insulin in poorly controlled adolescents with T1DM and overweight [60]. The results indicated that combining metformin with insulin therapy improved metabolic control in patients. However, the quality of metabolic control varied across studies due to differences in study design, inclusion and exclusion criteria, and methodologies. Adjunctive metformin therapy demonstrates a favorable impact on diabetes management, as well as in the prevention of cardiovascular complications with a minimal risk of adverse effects. The review also provides recommendations for further investigation of the research findings and their clinical implications [60].

In recent decades, there has been a notable rise in the prevalence and incidence of gout, with rates reaching 11–13% and 0.4% respectively among individuals aged over 80. Age-related decline in kidney function, changes in drug distribution, and a higher occurrence of other health conditions, including obesity, have notable implications for the safe and efficient pharmacological management of gout [61,62]. In a literature review, Patil et al. provide a concise summary of in vivo experimental models of gouty arthritis, focusing on evaluating the hypouricemic, anti-inflammatory, and renal protective effects of test compounds. They delve into specific evaluation parameters to offer a detailed perspective [61].

The escalating challenge of obesity underscores the necessity for a deeper comprehension of its pathophysiology, particularly concerning adipose tissue [[63], [64], [65], [66], [67], [68], [69], [70]]. Animal models of obesity play a pivotal role in exploring potential mechanisms and implications associated with this condition. The utilization of animal models in obesity research is important for exploring potential mechanisms and implications of the condition. However, it is essential to acknowledge and consider various factors, including anatomical and pathophysiological differences among species, when interpreting preclinical results [71]. Examples of conflicting results among preclinical models and human physiological studies include the natural history of obesity development, the presence of hypoxia in the adipose tissue of obese animals, the adipose tissue browning, endocrine function and fibrosis. Lempesis et al. summarized the similarities and differences between rodent models and humans, factors that should be duly considered in research studies focused on obesity [71].

Finally, this Special Issue features four research highlights on recent significant studies in metabolism [[72], [73], [74], [75]]. These highlights comment on recent studies on the mitochondrial quality control for promoting longevity [76], the action of manganese ions as a messenger to regulate serum lipid levels [77], the temporal and spatial dynamics of the immune response following myocardial infarction [78], and the role of apoptotic cells in supporting immune homeostasis by inhibiting Th17 cell differentiation [79].

In conclusion, we hope this Special Issue, featuring manuscripts from our esteemed colleagues, may advance the understanding of recent research in clinical metabolomics and metabolism. We sincerely believe these contributions could serve as valuable resources for readers and future investigators, aiding in the exploration of new research directions for the discovery and development of diagnostic, prognostic, and predictive biomarkers.

## Funding

This work did not receive any specific grant from funding agencies in the public, commercial or not-for-profit sectors.

## Conflicts of interest

The authors declare no conflict of interest.
